# Inhibiting Importin 4-mediated nuclear import of CEBPD enhances chemosensitivity by repression of PRKDC-driven DNA damage repair in cervical cancer

**DOI:** 10.1038/s41388-020-1384-3

**Published:** 2020-07-13

**Authors:** Yang Zhou, Fei Liu, Qinyang Xu, Bikang Yang, Xiao Li, Shuheng Jiang, Lipeng Hu, Xueli Zhang, Lili Zhu, Qing Li, Xiaolu Zhu, Hongfang Shao, Miao Dai, Yifei Shen, Bo Ni, Shuai Wang, Zhigang Zhang, Yincheng Teng

**Affiliations:** 1 Department of Gynecology and Obstetrics, Shanghai Jiao Tong University Affiliated Sixth People’s Hospital, 600 Yishan Road, Shanghai, 200233 PR China; 2 Department of Gynecology and Obstetrics, Shanghai Eighth People’s Hospital, Affiliated to Jiangsu University, Shanghai, 200233 PR China; 3 Global Clinical Medical Affairs (GCMA), Shanghai Henlius Biotech, Inc. 7/F, Innov Tower, Zone A, No.1801 HongMei Rd. Xuhui District, Shanghai, 200233 PR China; 4 State Key Laboratory for Oncogenes and Related Genes, Shanghai Cancer Institute, Shanghai Jiao Tong University, Shanghai, PR China; 5 Center of Reproductive Medicine, Shanghai Jiao Tong University Affiliated Sixth People’s Hospital, 600 Yishan Road, Shanghai, 200233 PR China; 6 Department of Gynecologic Oncology, Hunan Cancer Hospital, The Affiliated Cancer Hospital of Xiangya School of Medicine, Central South University, Changsha, PR China; 7 Department of Orthopedics, Shanghai East Hospital, School of Medicine, Shanghai Tongji University, Shanghai, 200120 PR China; 8 Department of Gastrointestinal Surgery, Ren Ji Hospital, School of Medicine, Shanghai Jiao Tong University, Shanghai, 200217 PR China; 9 Jacobi medical center, bronx, New York, NY 10461 USA

**Keywords:** Cancer therapeutic resistance, Cervical cancer

## Abstract

Cervical cancer (CC) remains highest in the mortality of female reproductive system cancers, while cisplatin (CDDP) resistance is the one of main reasons for the lethality. Preceding evidence has supported that karyopherins are associated with chemoresistance. In this study, we simultaneously compared CDDP-incomplete responders with CDDP-complete responders of CC patients and CDDP‐insensitive CC cell lines with CDDP‐sensitive group. We finally identified that DNA-PKcs (PRKDC) was related to CDDP sensitivity after overlapping in CC sample tissues and CC cell lines. Further functional assay revealed that targeting PRKDC by shRNA and NU7026 (specific PRKDC inhibitor) could enhance CDDP sensitivity in vitro and in vivo, which was mediated by impairing DNA damage repair pathway in CC. Mechanistically, we found that PRKDC was transcriptionally upregulated by CCAAT/enhancer-binding protein delta (CEBPD), while intriguingly, CDDP treatment strengthened the transcriptional activity of CEBPD to PRKDC. We further disclosed that Importin 4 (IPO4) augmented the nuclear translocation of CEBPD through nuclear localization signals (NLS) to activate PRKDC-mediated DNA damage repair in response to CDDP. Moreover, we demonstrated that IPO4 and CEBPD knockdown improved CDDP-induced cytotoxicity in vitro and in vivo. Together, we shed the novel insight into the role of IPO4 in chemosensitivity and provide a clinical translational potential to enhance CC chemosensitivity since the IPO4-CEBPD-PRKDC axis is actionable via NU7026 (PRKDC inhibitor) or targeting IPO4 in combination with CDDP.

## Introduction

Cervical cancer (CC) still ranks the first place in both the incidence and mortality of female reproductive system cancers despite progress in vaccine prevention, screening, and treatment according to the 2018 GLOBACAN estimates [1] and Systematic Analysis for the Global Burden of Disease Study in 2019 [2]. Thus, we need to be aware that there still lack effective strategies for CC treatment.

Platinum-based chemotherapy is currently employed as the standard strategy for advanced or recurrent CC patients [3], while cis-dichlorodiamineplatine (CDDP), a crosslink-inducing DNA-damaging agent [4], has been recommended as the preferred first-line single-agent for advanced CC (stages IB3, II, III, and IVA) [5–7]. However, the reported CDDP response rates are only 20 to 30% [8, 9] since intrinsic (increasing DNA repair) or acquired resistance (drug-induced) developed and thus resulted in chemotherapeutic failure in the long-term chemotherapy [10]. Accordingly, enhancing CDDP sensitivity is determinant to the chemotherapeutic outcome. To improve the sensitivity of CDDP, the following strategies have been proposed: (1) explore CDDP-combination alternatives; (2) exploit new platinum drugs [11, 12]; (3) improve CDDP delivery to cancers [13]; (4) specifically target CDDP resistance mechanisms. Therefore, there is a great unmet need to identify the underlying mechanisms of CDDP resistance to develop novel targets or CDDP-combination agents to enhance CDDP sensitivity.

Karyopherins, including importins and exportins, are known to recognize and bind to cargos through nuclear localization signal (NLS), and then utilize RanGTP for active transport across the nuclear membrane through the nuclear pore complex [14]. The cargos include transcription factors, splicing factors and other proteins [15–17]. Furthermore, emerging evidence has supported that overexpressed karyopherins result in the mislocalization of key mediators and associate with the tumorigenesis and chemoresistance in multiple tumors [18–24]. Thus, targeting karyopherins might be a promising chemotherapeutic strategy in cancers.

In this study, we simultaneously compared CDDP-incomplete responders with CDDP-complete responders of CC patients from Cancer Genome Atlas (TCGA) dataset and CDDP‐insensitive CC cell lines with CDDP‐sensitive group through Genomics of Drugs Sensitivity in Cancer (GDSC) dataset. We finally identified that PRKDC was related to CDDP sensitivity after overlapping in CC sample tissues and CC cell lines. As a critical component of DNA damage repair machinery [25], PRKDC is known to be associated with chemoresistance and radioresistance in some tumors [26, 27]. And our functional assays showed that knockdown of PRKDC enhanced CDDP sensitivity in CC both in vitro and in vivo, which was mediated by impairing DNA damage repair pathway. Mechanistically, we further discovered that PRKDC was upregulated by transcription factor CDDP-responsive gene (CEBPD). Interestingly, we uncovered that CDDP could strengthen the transcriptional activity of CEBPD to the promoter regions of PRKDC. Furthermore, we identified that the nuclear translocation of CEBPD was mainly mediated by importin 4 (IPO4) through NLS and was protected from degradation by IPO4.

Taken together, we demonstrated that IPO4 physically bound to CEBPD and augmented its nuclear translocation in response to CDDP treatment, which further enhanced PRKDC transcription to render CDDP resistance.

## Results

### PRKDC is associated with CDDP resistance and predicts poor prognosis in CC

**Fig. 1:**
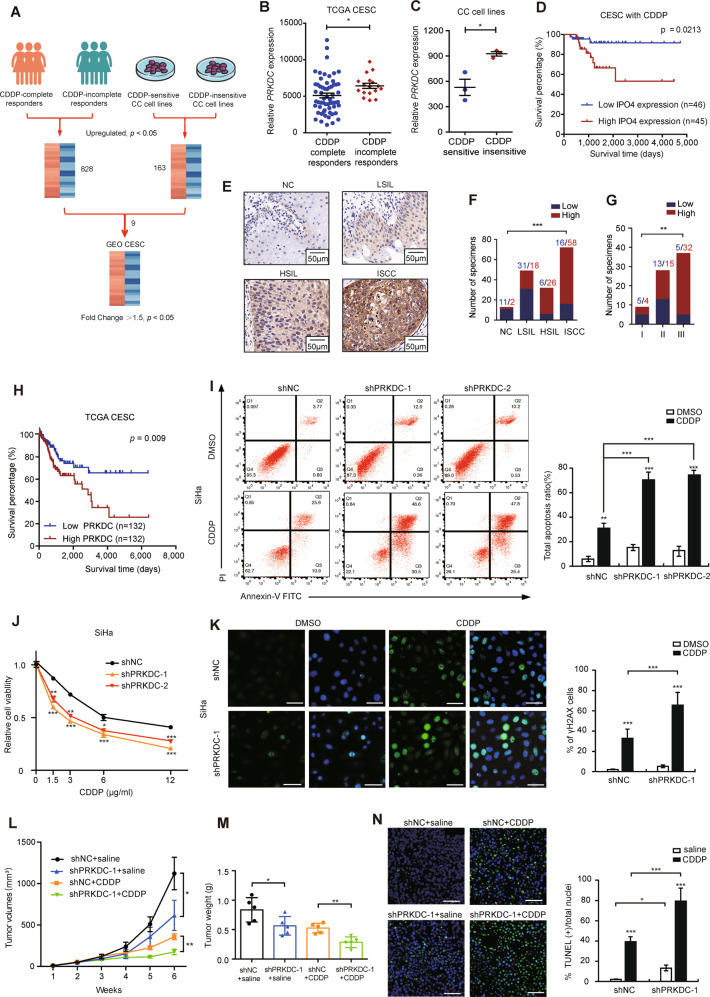
PRKDC predicts poor survival in CC patients and confers CDDP resistance. **a** 828 upregulated expression of genes in CDDP-incomplete responders compared with CDDP-complete responders in TCGA dataset. Fold changeå 1.25, *p* < 0.05. 163 upregulated expression of genes in CDDP-insensitive CC (higher IC50) cell lines compared with CDDP-sensitive CC cell lines (lower IC50) with fold change (FC) > 1.25 and *P* < 0.05. A total of 9 genes were identified after overlapping in CC tissue samples and CC cell lines, Next, these 9 genes were further analyzed in CC tissues and normal tissues from GSE9750 datasets. Fold changeå 1.5, *p* < 0.05. (Student’s *t* test). **b** PRKDC expression in tissue samples from CC patients who are complete responders (*n* = 59) or incomplete responders (*n* = 17) to CDDP. Data were presented as mean ± SD. (Student’s *t* test). **c** PRKDC expression in CDDP-insensitive CC cell lines (*n* = 3) and CDDP-sensitive CC cell lines (*n* = 3). Data were presented as mean ± SD. (Student’s *t* test). **d** The survival curve for CC patients with CDDP treatment based on the expression of PRKDC from the TCGA dataset. **e** Representative images of the PRKDC immunoreactivity in NC, LSIL, HSIL, ISCC. (scale bar: 50 μm). NC normal tissue, ISIL low grade squamous intraepithelial lesion, HSIL high grade squamous intraepithelial lesion, ISCC invasive squamous cell carcinoma of the cervix. **f** The constituent ratio of PRKDC expression analyzed by IHC in NC (*n* = 13), LSIL (*n* = 49), HSIL (*n* = 32) and ISCC (*n* = 74). (one-way ANOVA test). **g** Statistical analysis of IHC staining results based on the expression level of PRKDC in stage I (*n* = 9), stage II (*n* = 28) and stage III (*n* = 37). (one-way ANOVA test). **h** Kaplan–Meier 5-year survival of CC patients in TCGA dataset. Patients with high PRKDC expression had a shorter overall survival (*p* = 0.0279). **i** Apoptosis assay staining the effect of 0.1%DMSO or 3 μg /ml CDDP for 24 h on the percentages of apoptotic SiHa cells. Data were presented as mean ± SD. (Student’s *t* test). **j** Cell viability assay showing the sensitivity to CDDP in SiHa cells stably expressed shNC and shPRKDCs in different concentration of CDDP. Data were presented as mean ± SD. (Student’s *t* test). **k** Immunofluorescence showing the DNA damage induced by CDDP (6 μg/ml, 1 h) in SiHa/shNC and shPRKDC-1 cells. Scale bar indicated 50 μm. Analysis of the percentage of cells with predominantly nuclear γH2AX. γH2AX is shown by green fluorescence, and the cell nuclei were stained with DAPI (blue fluorescence). *n* = 3 randomly chosen fields. Data were presented as mean ± SD. (Student’s *t* test). **l** SiHa cells stably expressing shNC and shPRKDC-1 were injected subcutaneously into 5-week-old nude female mice. When tumors born palpable (100 mm^3^), mice of shNC and shPRKDC-1 groups were intraperitoneally injected (i.p) with saline or CDDP (5 mg/kg) every 3 days, thus divided into the four groups (*n* = 5) (shNC+saline, shNC+CDDP, shPRKDC-1+saline, shPRKDC-1+CDDP). Tumors were excised from the mice and weighed after 7 weeks. Tumor volumes were measured with calipers every 7 days. scale bar: 1 cm. Values are mean ± SEM. (Student’s *t* test). **m** Tumor weight was shown as mean ± SD of shNC+saline, shNC+CDDP, shPRKDC-1+saline, shPRKDC-1+CDDP groups. (Student’s *t* test). **n** Representative images of TUNEL staining in xenograft tumors from four groups. DAPI-counterstained nuclei are in blue, and TUNEL is in green. Data were presented as mean ± SD. (Student’s *t* test). *p < 0.05, **p < 0.01 and ***p < 0.001.

To find out the genes related to CDDP resistance, we included patients from TCGA dataset as following criteria: (1) cervical squamous cell carcinoma; (2) with only CDDP chemotherapy; (3) sufficient follow-up information with CDDP response; (4) with mRNA data, then we divided patients with CDDP treatments into two groups according to their response to CDDP: complete responders (*n* = 59) and incomplete-responders (*n* = 17), corresponding to “complete response/remission” and “partial response/remission, stable diseases and progressive diseases”, respectively. We identified 828 upregulated expression of genes in incomplete-responders group. Meantime, we downloaded data of CC cell lines expression profiling assay from Gene Expression Omnibus database (GEO) (GSE9750). The information of CDDP IC50 value of CC cell lines was extracted from Genomics of Drugs Sensitivity in Cancer (GDSC, https://www.cancerrxgene.org). 6 CC cell lines were then divided into two groups, namely, CDDP‐insensitive group (SW756, SiHa, CaSki) and CDDP‐sensitive group (MS751, HT-3, C-33A) according to their IC50 value of CDDP, and we found 163 upregulated genes in CDDP‐insensitive group compared with CDDP‐sensitive group. A total of 9 genes were then identified after overlapping in CC sample tissues and CC cell lines, namely, ACTR2, OSMR, CDP, PEX3, PRKDC, ZFR, RASA2, TRAM2, SLC25A24. Next, these 9 genes were further analyzed in GSE9750, and only PRKDC were found to be upregulated in cancer tissues compared to the normal tissues (Fig. 1a–c). Furthermore, we analyzed the prognosis of PRKDC and found that PRKDC correlated with a poor overall survival in CC patients receiving CDDP treatment (Fig. 1d). Consistent with the mRNA expression (Supplementary Fig. S1a–f), the protein level of PRKDC was also much higher in CC tissues than that in normal tissues and increased with the advanced malignancy (Fig. 1e–g). Moreover, we found that the higher expression of PRKDC predicted a poor overall survival in CC patients (Fig. 1h).

### Reduced PRKDC enhances CDDP sensitivity via increasing DNA damage in vitro and in vivo

To further confirm whether PRKDC could affect CDDP sensitivity of CC, we first suppressed the expression of PRKDC by shRNA in both SiHa and C4I cells expressing relatively higher level of PRKDC (Supplementary Fig. S2a, b). As expected, knockdown of PRKDC significantly increased CDDP sensitivity in both SiHa and C4I cells (Fig. 1j and Supplementary Fig. S2c). Moreover, the apoptosis assay revealed that CDDP induced more apoptosis after silencing of PRKDC in both SiHa and C4I cells (Fig. 1i and Supplementary Fig. S2d). To determine whether the higher PRKDC was responsible for DNA damage repair in CC, we measured the expression of phosphorylated H2AX (γH2AX), a quantitative measurement of DNA damage [28]. The results showed that the amount of CDDP-induced γH2AX was noticeably higher in shPRKDCs than that in shRNA control (shNC) cells (Fig. 1k and Supplementary Fig. S2e), indicating that knockdown of PRKDC could induce more DNA damage. We further validated these results in a xenograft mouse model. SiHa cells stably expressing shPRKDC-1 and control shRNA were injected into the flank of 5-week-old nude mice. Treatment with CDDP significantly shrank the xenograft tumors both in shPRKDC-1 and shNC groups. In addition, knockdown of PRKDC significantly increased the CDDP-induced tumor suppression (Supplementary Fig. S3 and Fig. 1l, m). Furthermore, TUNEL assay revealed a higher CDDP-induced apoptosis rate in the PRKDC-knockdown xenografts (Fig. 1n). Meanwhile, shPRKDC alone could also increase CC apoptosis compared with shNC in vitro and in vivo. These results indicated that targeting PRKDC enhanced CDDP sensitivity of CC both in vitro and in vivo.

### NU7026 improves CDDP sensitivity via enhancing DNA damage in vitro and in vivo

**Fig. 2:**
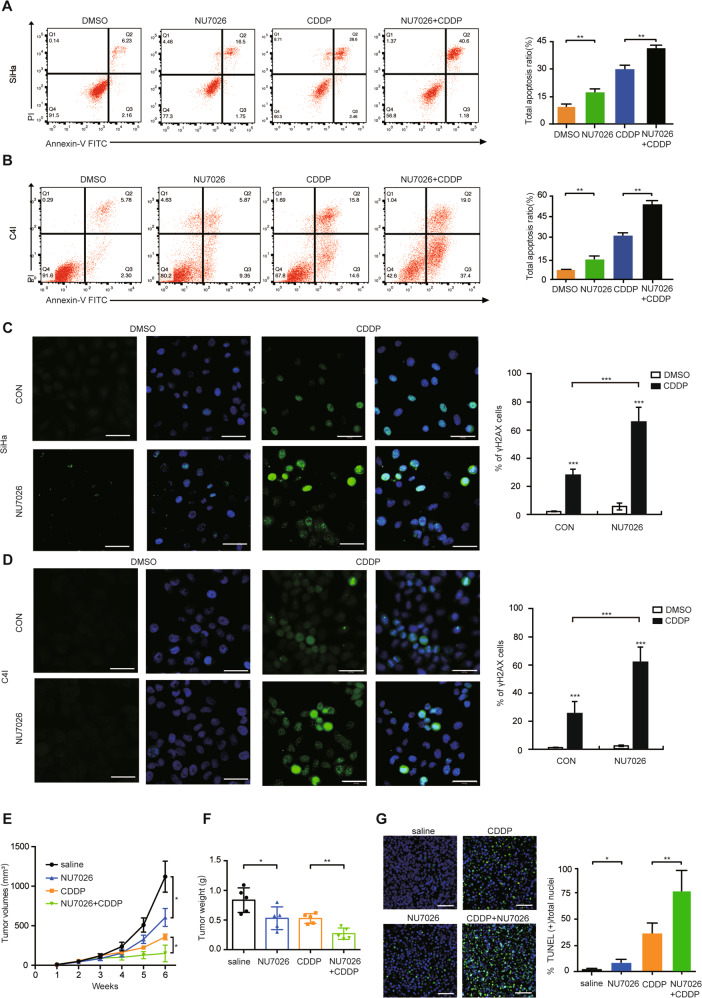
NU7026 enhances the sensitivity to CDDP in CC in vitro and in vivo. **a**, **b** Cell apoptosis assay for SiHa and C4I cells treating with 0.1%DMSO, NU7026 (2 μM), CDDP (3 μg/ml) or combination of NU7026 and CDDP for 24 h. Values are mean ± SD. (Student’s *t* test). **c, d** Representative immunofluorescence staining and analysis of the amount of γH2AX in SiHa and C4I cells treated with CDDP (6 μg/ml) for 1 hour. Scale bar indicated 50 μm. Values are mean ± SD. (Student’s *t* test). **e** Stable SiHa cells were injected into nude mice. When tumors became visible (100 mm^3^), mice were treated with saline, NU7026, CDDP with NU7026. Time-course of xenograft growth in four different groups (saline, CDDP, NU7026, NU7026 + CDDP) (*n* = 5) were measured with calipers every 7 days. Scale bars = 1 cm. Values are mean ± SEM. (Student’s *t* test). **f** Tumor weight of above four groups. Values are mean ± SD. (Student’s *t* test). **g** Representative images of TUNEL staining in xenograft tumors from four groups. Ratio of TUNEL positive cells. Scale bar indicated 100 μm. Values are mean ± SD. (Student’s *t* test). *p < 0.05, **p < 0.01 and ***p < 0.001.

Regarding to the clinical significance, we explored whether NU7026, the PRKDC specific inhibitor, could enhance CDDP sensitivity in CC in vitro and in vivo. As expected, we found that combined treatment with CDDP and NU7026 induced markedly more apoptosis (Fig. 2a, b) and DNA damage (Fig. 2c, d) than that treatment with CDDP alone in both SiHa and C4Icells, indicating that NU7026 could inhibit DNA damage repair, thus sensitized CC cells to DNA-damage chemotherapy. To further test the efficacy in vivo, CDDP combined with NU7026 were intraperitoneally injected into the nude mice every 3 days. Consistent with our in vitro findings, mice treated with the combination of CDDP and NU7026 exhibited the significantly smaller tumors (Supplementary Fig. S3 and Fig. 2e, f) and more apoptosis (Fig. 2g) compared with the control or single-agent treatment groups. In addition, NU7026 alone could also increase CC apoptosis compared with control in vitro and in vivo. Collectively, these data revealed that NU7026 and CDDP could inhibit synergistically the CC cell proliferation in vitro and in vivo, suggesting that NU7026 and CDDP combination could provide an additional benefit of chemotherapy in CC.

### CDDP strengthens the transcriptional activity of CEBPD to *PRKDC* promoters

**Fig. 3:**
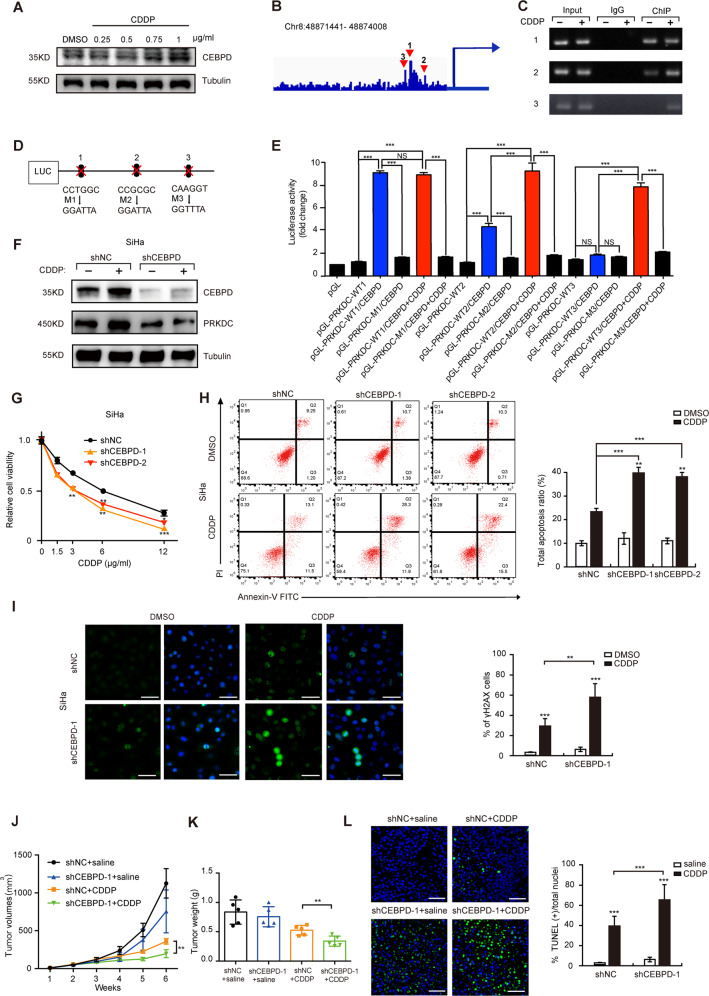
CDDP enhances the transcriptional activity of CEBPD to PRKDC promoters and CEBPD attenuates CDDP sensitivity in vitro and in vivo. **a** Western blot analysis for CEBPD level was conducted with lysates harvested from different concentration of CDDP-treated SiHa for 24 h. **b** The potential PRKDC binding sites (1, 2, 3) in SiHa cell lines were predicted by the program IGV_2.5.3. **c** A ChIP assay was used to verify the potential CEBPD co-binding sites in the PRKDC promoter regions in SiHa cell with or without CDDP treatment (1 μg/ml, 24 h). Sonicated input DNA and IgG were used as controls. **d** Diagram of 3 binding site mutant plasmids including mutant 1(M1), mutant 2 (M2) and mutant 3 (M3). **e** Luciferase activities in luciferase reporter plasmids containing wild-type and M1, M2 and M3 PRKDC promoters were indicated in SiHa cell with or without CDDP treatment (1 μg/ml, 24 h). The pGL3 promotor vector without enhancer sequence was used as control construct. Values are mean ± SD. (Student’s *t* test). **f** Knockdown of CEBPD and control shRNA SiHa cell were treated with 1 μg/ml CDDP for 24 h followed by western blotting. (Student’s *t* test). **g** Cell viability assay in SiHa/shNC and shCEBPD-1 cells at different concentration of CDDP. Values are mean ± SD. (Student’s *t* test). **h** Apoptotic assay in SiHa/shNC and shCEBPD-1 cells after treatment with 0.1%DMSO or 3 μg/ml CDDP for 24 h. Values are mean ± SD. (Student’s *t* test). **i** γH2AX assay in SiHa/shNC and shCEBPD-1 cells with CDDP treatment (6 μg/ml, 1 h). Refer to Fig. 1j. **j** Tumor volumes of xenograft tumors from SiHa/shNC+saline, shNC+CDDP, shCEBPD-1+saline, shCEBPD-1+CDDP groups (*n* = 5). Scale bars = 1 cm. groups of shNC+saline and shNC+CDDP were taken the same as previous mentioned. Values are mean ± SEM. (Student’s *t* test). Refer to Fig. 1l. **k** Tumor weight in above four groups. (Student’s *t* test). **l** TUNEL assay in above four groups. Scale bar indicated 100 μm. (Student’s *t* test). NS no significance, *p < 0.05, **p < 0.01 and ***p < 0.001.

Next, we further explored the mechanism how the expression of PRKDC was upregulated. CCAAT/enhancer-binding protein delta (CEBPD) was reported to be inducible by CDDP [29] and led to chemoresistance in bladder cancer [30, 31]. Coincident with the previous studies, we also demonstrated that CDDP induced the expression of CEBPD in a dose manner in CC (Fig. 3a). Furthermore, both JASPAR (http://jaspar.genereg.net/) and GCBI (https://www.gcbi.com.cn/gclib/html/index) websites showed the CEBPD-binding elements in the *PRKDC* promoters. Therefore, we reasonably hypothesized that CEBPD might upregulate PRKDC. We then predicted 3 potential CEBPD-binding sites on *PRKDC* promoters (1, 2, 3) in SiHa cell by the program IGV_2.5.3 (Fig. 3b) [32]. As displayed in Fig. 3c, the ChIP results showed that CEBPD-binding sites of 1 and 2 were detected at the *PRKDC* promoters. Interestingly, we found the binding site of 3 was activated following CDDP treatment and the binding affinity of binding site 2 was markedly enhanced. Moreover, luciferase reporter (Fig. 3d, e) also verified that *PRKDC* was the target gene of CEBPD and CDDP treatment augmented the transcriptional activation of CEBPD to *PRKDC* promoters. To provide the additional evidence, we confirmed the results by western blotting (Fig. 3f). Overall, we demonstrated that CDDP could enhance the transcriptional activity of CEBPD to *PRKDC* promoters.

### CEBPD knockdown drives the CDDP-induced DNA damage in vitro and in vivo

To further investigate whether CEBPD could influence CDDP sensitivity of CC, we down-regulated the expression of CEBPD in CC cells. The results showed that CEBPD knockdown enhanced cell apoptosis, cell toxicity and DNA damage in response to CDDP in both SiHa and C4I cells (Fig. 3g–i and Supplementary Fig. S4a–c). To validate these findings *in vivo*, we injected SiHa cells stably expressing shCEBPD-1 and control shRNA into the 5-week-old nude mice. We found that knockdown of CEBPD exhibited more CDDP-induced reduction of tumor growth and enhancement of cell apoptosis (Supplementary Fig. S3 and Fig. 3j–l).

### IPO4 augments CEBPD nuclear translocation by NLS in response to CDDP and regulates CEBPD stability

**Fig. 4:**
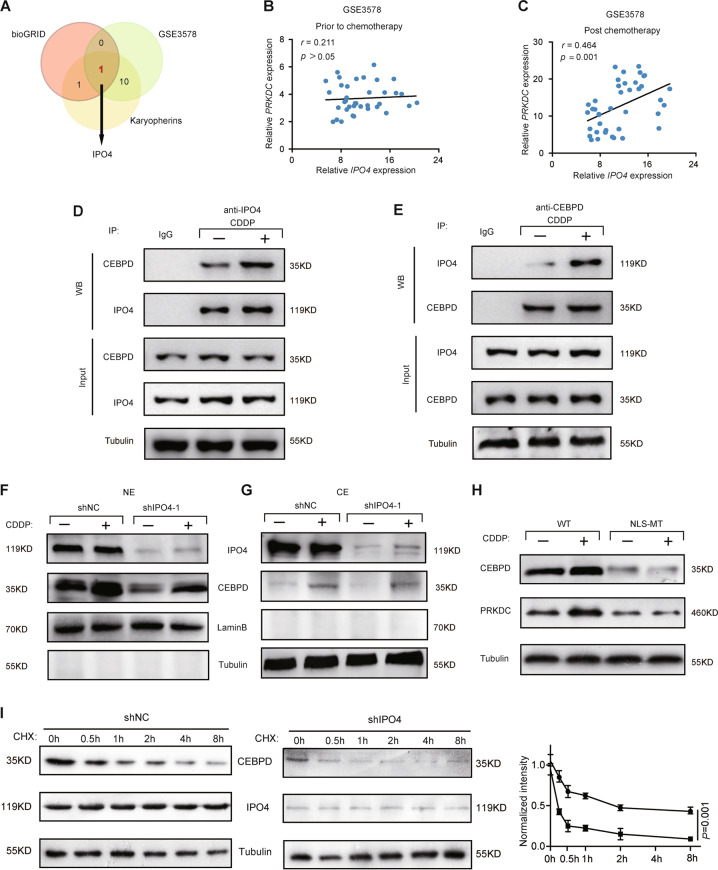
IPO4 augments nuclear translocation of CEBPD in response to CDDP by NLS. **a** The Venn diagram of screened karyopherins in CC. We first analyzed 25 karyopherins in the chemotherapy database of CC (GSE3578), and found that 10 karyopherins fitting the filtering criteria that the correlation between karyopherins and PRKDC was increased after chemotherapy. Next, we identified only IPO4, among above 10 karyopherins, could interact with CEBPD through bioGRID (https: //thebiogrid.org). **b**, **c** Correlation of IPO4 with PRKDC prior to/after the chemotherapy in GSE3578 dataset. (Spearman rank correlation test). **d**, **e** Siha cells were transfected with flag-tagged CEBPD expression constructs and treated with CDDP (1 μg/ml) for 24 h. Lysates were immunoprecipitated (IP) with anti-CEBPD antibody, anti-IPO4 antibody and IgG control. Interaction of IPO4 and CEBPD proteins and quantitative analysis in triplicate were performed. **f**, **g** SiHa cells were stably transfected with shRNA against IPO4 with shRNA as control. CDDP (1 μg/ml) was added 24 h later, and cells were incubated for another 24 h before preparation of cytoplasmic (CE) or nuclear (NE) cell extracts. Data shown are mean ± SD. Tubulin and Lamin B were used as internal controls for CE and NE. **h** SiHa cell was co-transfected CEBPD NLS-deficient mutant (p.195-222del) or wild-type plasmids with siRNA-CEBPD, while siRNA-CEBPD target at 3’UTR (target sequences: sense: 5‘-CAGCUAAGGUACAUUUGUATT-3′, anti-sense: 5‘-UACAAAUGUACCUUAGCUGTT-3′), which did not interfere with the expression of wild-type and NLS-mutant plasmids. The impact of CEBPD NLS-deficient mutant plasmid on PRKDC level in response to CDDP was detected by western blotting. **i** SiHa cells expressing shNC or shIPO4 were treated with CHX (100 mg/ml) for the indicated time points, 0, 0,5, 1, 2, 4, 8 h respectively. SiHa cells was transfected with either control shRNA or shIPO4 for 48 h, followed by CHX (100 mg/ml) treatment for the indicated times. The cell extracts were detected by WB.

To identify which karyopherin could facilitate the nuclear transport of CDDP-induced CEBPD to further enhance the transcription of PRKDC, we first analyzed karyopherins in the chemotherapy database of CC (GSE3578) and set up the filtering criteria: the correlation between karyopherins and PRKDC was increased after chemotherapy. The results showed that 10 karyopherins fitted the criteria (Supplementary Table 1). To further identify which karyopherin could directly interact with CEBPD and import it into the nucleus, we searched for CEBPD potential interaction partners in bioGRID (https: //thebiogrid.org), which is used to predict protein-to-protein interaction, and found importin 4 (IPO4) might be an interaction partner for CEBPD (Fig. 4a–c). Then we performed co-IP assay in CC cells and confirmed the direct physical interaction between IPO4 and CEBPD. Intriguingly, the strength of interaction was enhanced by treatment with CDDP (Fig. 4d, e). Furthermore, the co-localization of IPO4 and CEBPD was further confirmed by immunofluorescence (Supplementary Fig. S5). To further prove that IPO4 is a *bona fide* importer for CEBPD nuclear translocation, we suppressed the expression of IPO4 in SiHa and found that knockdown of IPO4 significantly reduced the nuclear localization of CEBPD both before and after CDDP treatment (Fig. 4f, g).

Previous study has demonstrated that CEBPD lacking the IPO4 interaction domain (amino acids 195–222) could not enter the nucleus [33]. To further examine whether CEBPD NLS-deficient mutant could affect PRKDC level in response to CDDP, we co-transfected CEBPD NLS-deficient mutant (p.195-222del) or wild-type plasmids and siRNA-CEBPD (target at 3’untranslated region (3’UTR), which does not interfere with the expression of either plasmid) in SiHa cell. We found that the expression of CEBPD NLS-deficient MUT was decreased compared with wild type, and was not induced by CDDP, meanwhile, the expression of PRKDC was decreased since the ineffective transcriptional activity of CBEPD NLS-deficient MUT. In addition, CBEPD-MUT could not enhance the expression of PRKDC in response to CDDP (Fig. 4h), which indicated that CEBPD-MUT abrogated its transcriptional activity for PRKDC in response to CDDP. These results collectively suggested that the NLS sequence (amino acids 195–222) of CEBPD was required for IPO4-mediated CEBPD nuclear transport to enhance the expression of PRKDC in response to CDDP.

Previous study [34] has reported that nuclear localization signal-inactivated mutant of RelB was unstable in the cells. Similarly, we also found the decreased expression of CEBPD NLS-deficient mutant plasmid and declining cytoplasmic accumulation of CEBPD after interfering by IPO4, we then speculated whether IPO4 could regulate CEBPD stability and shIPO4 could accelerate its degradation. To verify our hypothesis, we examined the effect of IPO4 interfering on the stability of endogenous CEBPD protein in the presence of the inhibitor of protein translation, cycloheximide (CHX) after 0, 0.5, 1, 2, 4, 8 h. The results indicated that CEBPD protein was obviously degraded more rapidly in IPO4-knockdown cells compared with control cells (Fig. 4i). These data collectively suggested that IPO4 interacted with and regulated CEBPD stability in CC cell.

Together, we demonstrated that CDDP could not only induce CEBPD expression but also enhance the physical interaction of IPO4 and CEBPD, thus facilitate the IPO4-mediated nuclear import of CEBPD by NLS.

### IPO4 attenuates CDDP sensitivity and predicts poor prognosis in CC patients treated with CDDP

**Fig. 5:**
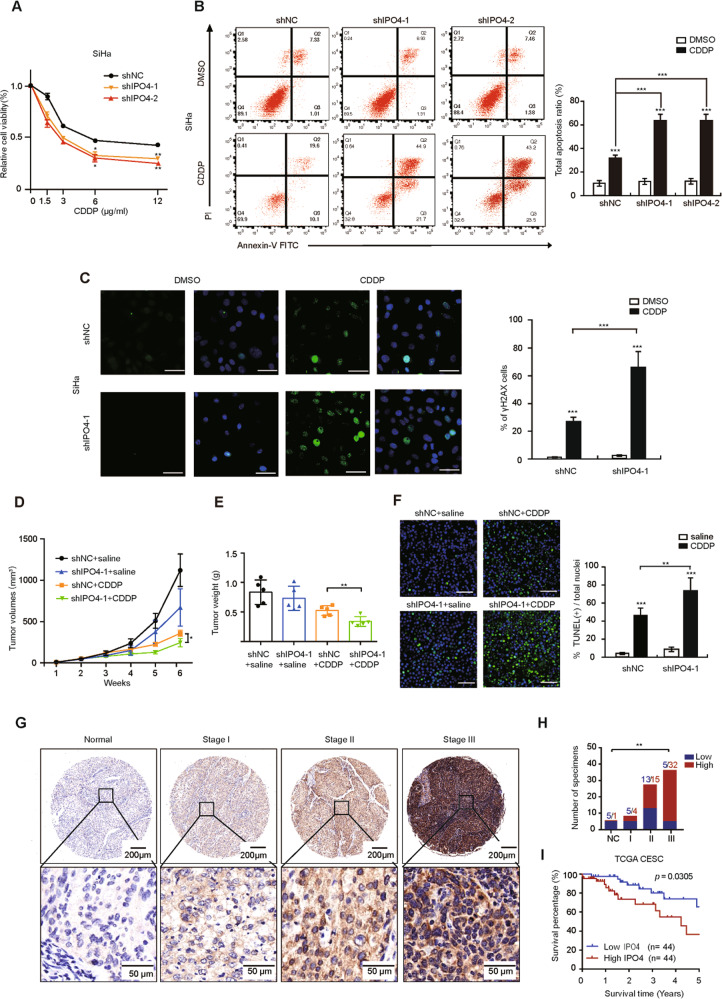
IPO4 attenuates CDDP sensitivity and correlates with poor prognosis in CC patients. **a** SiHa/shNC and shIPO4-1cell viability were treated with different concentrations of CDDP. Refer to Fig. 1i. **b** Apoptotic assay in SiHa/shNC and shIPO4-1 cells. Refer to Fig. 1h. **c** γH2AX assay in SiHa/shNC and shIPO4-1 cells. Refer to Fig. 1j. **d** Tumor volumes of xenograft tumors from SiHa /shNC+saline, shNC+CDDP, shIPO4-1+saline, shIPO4-1+CDDP groups (*n* = 5). Scale bars = 1 cm. Control groups of shNC+saline and shNC+CDDP were taken the same. Values are mean ± SEM. (Student’s *t* test). **e** Tumor weight in above four groups. Scale bar indicated 1 cm. (Student’s *t* test). **f** TUNEL assay in above four groups. Scale bar indicated 100 μm. Values are mean ± SD. (Student’s *t* test). **g** Representative photographs of the IPO4 immunoreactivity in normal tissue, stage I, II, III in CC tissues (scale bar: 200 μm, 50 μm). **h** Statistical analysis of IHC staining results based on the protein level of IPO4 in normal and tumor tissues (Norma tissue: *n* = 6; stage I: *n* = 9; stage II: *n* = 28; stage III: *n* = 37). (one-way ANOVA test). **i** Higher IPO4 correlates with poor overall survival curves of CC patients from TCGA datasets. *p < 0.05, **p < 0.01 and ***p < 0.001.

To validate whether IPO4 could regulate CDDP sensitivity of CC, we silenced the expression of IPO4 in CC cells and found that knockdown IPO4 increased CDDP-induced cytotoxicity (Fig. 5a and Supplementary Fig. S6a), the apoptosis rate (Fig. 5b and Supplementary Fig. S6b) and DNA damage (Fig. 5c and Supplementary Fig. S6c) both in SiHa and C4I cells. In addition, Siha cells with stable expression of shIPO4-1 resulted in smaller tumors (Supplementary Fig. S3 and Fig. 5d, e) and more CDDP-induced apoptosis (Fig. 5f) compared with the control group. To further confirm the clinical relevance of above findings, we then performed immunohistochemistry staining (IHC) for IPO4 in normal cervical tissues (*n* = 6), stage I (*n* = 9), stage II (*n* = 28) and stage III (*n* = 37) CC tissues and found the protein level of IPO4 was much higher in CC than that in normal tissues and increased with the advanced malignancy (Fig. 5g, h), in agreement with the results of mRNA (Supplementary Fig. S7a–c). In addition, we further found that higher IPO4 was correlated with worse overall survival in CC patients (Fig. 5i). Together, we demonstrated that inhibition of IPO4, a nuclear import protein, potentiates CDDP sensitivity and the higher expression of IPO4 was correlated with poor prognosis in CC patients treated with CDDP.

## Discussion

**Fig. 6:**
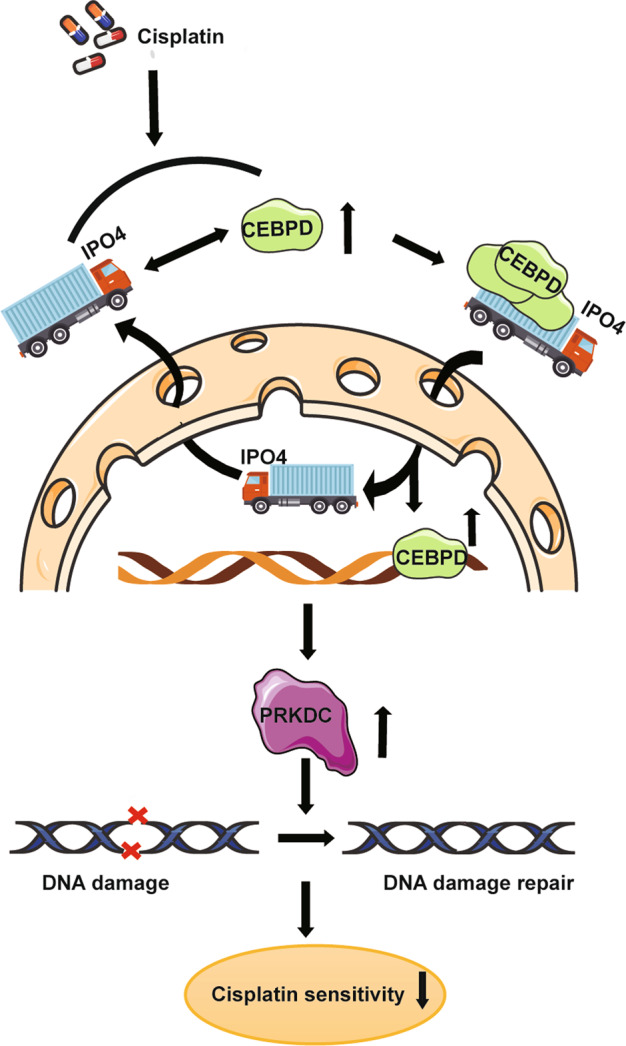
The schematic diagram of IPO4-CEBPD-PRKDC axis in modulating CC chemosensitivity. Upon the treatment of CDDP, CEBPD is induced and the interaction of CEBPD and IPO4 is strengthened. Importin 4 (IPO4) augment the nuclear translocation of CEBPD, and CEBPD transcriptionally upregulated PRKDC, which lead to DNA damage repair and thus blunted CDDP sensitivity.

In this study, we demonstrated that CDDP treatment-induced CEBPD expression and further strengthened the interaction between CEBPD and IPO4. Thus IPO4-dependent nuclear translocation of CEBPD was enhanced and expression of CEBPD-driven PRKDC was increased. Finally, PRKDC-mediated DNA damage repair was accomplished to inhibit chemosensitivity in CC (Fig. 6).

Aberrant expression/activation PRKDC has been reported to be associated with chemoresistance in a variety of tumors including glioma, oral squamous cell carcinoma, ovarian cancer and breast cancer due to its function in DNA damage repair [26, 27, 35–37]. While breaking DNA damage repair by silencing PRKDC has been reported to improve chemosensitivity [26, 27, 35–37]. However, the role of PRKDC in CDDP sensitivity in CC has never been investigated. In our study, we demonstrated that genetic or pharmacologic inhibition of PRKDC could improve CC chemosensitivity. Besides, our results indicated that PRKDC could serve as a predictor for CDDP sensitivity in advanced/recurrent CC patients, which was beneficial to guide individual chemotherapy.

Mechanistically, we revealed that PRKDC was transcriptionally activated by CEBPD. Moreover, our study highlighted that CDDP enhanced the transcriptional activity of CEBPD to PRKDC promoters. These findings provide an explanation as why acquired chemoresistance develops and CC patients become desensitized to CDDP chemotherapy. CEBPD is used to be labeled as a “tumor suppressor” [38]. However, the oncogenic role of CEBPD under certain conditions becomes more evident [39, 40]. Herein, we confirmed that CEBPD expression was induced by CDDP in CC. Additionally, silencing CEBPD enhanced CDDP sensitivity in vitro and in vivo. Nevertheless, it’s rather difficult to target the transcriptional factor directly. Thus, we turn to target the nuclear transport of CEBPD instead.

Accumulating evidence has shown that upregulated karyopherins were implicated in the carcinogenesis in multiple tumors [41]. Recent studies have revealed the oncogenic role of IPO3/KPNA4 in the head and oncogenic neck squamous cell carcinomas [42] and the potential target of KPNA2 in gallbladder cancer [43]. In addition, emerging studies have revealed that exportin-1(XPO1) is involved in chemoresistance in many cancers, and XPO1 inhibitor, Selinexor, has been gradually accepted as an effective alternative to overcome chemoresistance [21, 44, 45]. A very recent study has demonstrated that importin-β/karyopherin-β1 modulated taxane sensitivity in cancer cells [46]. Nevertheless, the association between importin β and chemoresistance remains poorly understood. IPO4, as an importin-β family protein, has rarely been reported in cancers and chemoresistance. There was only one paper that reported IPO4 functions as a driving force in human primary gastric cancer [47]. Until now, existing studies have shown that IPO4 is responsible for the nuclear translocation of key mediators, such as DNA repair protein FANCD2, ribosomal protein rpS3α, vitamin D receptor. In our study, we found that IPO4 physically interacted with CEBPD and facilitated nuclear transport of CEBPD. More interestingly, upon CDDP treatment, the IPO4-CEBPD interaction was strengthened and the nuclear translocation of CEBPD was increased. Besides, we found that NLS-mutant CEBPD was unstable and IPO4 protected CEBPD from degradation and slowed down its turnover. This finding was in line with previous evidence that the stability of nuclear protein depended on its nuclear distribution, which was often mediated by karyopherins. One study has reported that nuclear localization signal-inactivated mutant of RelB was unstable in the cells, and associated with the affinity to importin-α5 [34]. In addition, nuclear Notch-1 was reported to be degraded after targeting XPO1 in pancreatic ductal adenocarcinoma [48].

To our best of knowledge, it is the first time that IPO4 is demonstrated to be correlated with the tumorigenesis and chemoresistance in CC. Accordingly, we provide more evidence to in-depth understand the function of importin β in modulating CC tumorigenesis and chemoresistance and may be extrapolated to other cancer types.

Since cancer cells seem to be vulnerable to the karyopherin inhibitors than non-cancer cells due to increased proliferative and metabolic demands [49, 50], specific karyopherins (importin/exportin) inhibitors alone or in combination with other chemotherapy exhibit its potential as a promising chemotherapeutic strategy. However, the development of inhibitors targeting nuclear import protein lags behind that of nuclear export inhibitors up to now [14]. We failed to confirm the efficacy of pharmacological inhibition of IPO4 in our study since there is no specific inhibitor for IPO4. Therefore, developing the specific inhibitor for IPO4 should be the future research orientation in overcoming chemoresistance.

Another limitation we need to mention was that although we identified that only IPO4 could augment nuclear import of CEBPD, we could not fully exclude the possibility that other karyopherins may also mediate the nuclear translocation of CEBPD since karyopherins are supposed to share common cargo [51]. But what we need to point was that among all the 25 karyopherins, we only found that IPO4 was higher in CC, and associated with poor survival in CC patients, and more importantly, its correlation with PRKDC was enhanced after chemotherapy. Thus we focused on IPO4 rather than other karyopherins, since the more “trucks“, the higher ability of transporting cargo protein of “CEBPD” by IPO4 in CC. Nevertheless, our team will further carry on the possibilities of other karyopherin-CEBPD interaction in CC.

Overall, we proposed a novel strategy of targeting IPO4 for overcoming CC chemoresistance, and unveiled a new insight into the mechanism of IPO4-CEBPD-PRKDC axis in CC chemoresistance, which, in turn, blocking IPO4-CEBPD-PRKDC axis by either NU7026 or targeting IPO4 might be a promising chemotherapeutic strategy for CC.

## Materials and methods

### Cell culture, transfections and plasmids

SiHa, HeLa, Ms751, CaSki, C4I, C33A were all obtained from Shanghai Cancer Institute, School of Medicine, Shanghai Jiao Tong University. All cells were grown in Dulbecco’s modified Eagle’s medium containing 10% fetal bovine serum (FBS) and 1% penicillin/streptomycin (P/S) as previously described [52]. And they were cultivated at 37 °C in a 5% CO_2_ atmosphere. All cell lines were performed verification in January 2017 and regularly tested (every 4 months) to make certain of mycoplasma negative by Shanghai Cancer Institute. Gene Pharma (Shanghai, China) assisted in the design and production of shRNA. shPRKDCs, shCEBPDs, shIPO4s and shscramble were generated by PLKO-puro lentiviral plasmids (Sigma). SiHa and C4I cells were infected with lentivirus and selected for 5 μg/ml puromycin (Gibco, A1113802) resistance. The efficiency of the knockdown was tested by western blot. Following are the sequences: shPRKDC, 5′-GATCCGCCATCCCTTATAG

GTTAATATCTCGAGATATTAACCTATAAGGGATGGTTTTTTG3′; shCEBPD5′CCGGGCCGACCTCTTCAACAGCAATCTCGAGATTGCTGTTGAAGAGGTCGGCTTTTT-3′; shIPO4,5′-GATCCGCTATTCAAGGGAGGTAATCTCGAG

ATTACCTCCCTTGAATAGCTTTTTT-3′. SiRNA targeting against CEBPD was purchased from Gene Pharma (Shanghai, China) (sense: 5‘-CAGCUAAGGUACAUUUGUATT-3′, anti-sense: 5‘-UACAAAUGUACCUUAGCUGTT-3′). SiRNA transfection was performed using LipofectamineRNAiMAX (13778150, Invitrogen, Carlsbad, CA, USA). The CEBPD wild type and CEBPD NLS-mutant plasmid (p.195-222del) (deficient sequence: CGGCAGCGGCGCGAGCGCAACAACATCGCCGTGCGCAAGAGCCGCGACAAGGCCAAGCGGCGCAACCAGGAGATGCAGCAGAAG) were generated by genomeditech (Shanghai, China). And cycloheximide (CHX) was purchased from MCE.

### Cell CDDP sensitivity assay

CDDP sensitivity assay was performed by cell counting kit-8 (CCK-8, Dojindo Molecular Technologies, Japan), and SiHa and C4Icells were transferred in 96-well plates at a density of 3000 cells per well. Target cells were pretreated with NU7026 (S2893, Selleck) for 24 h. Different concentration of CDDP (MCE, shanghai, China) was diluted in dimethyl sulfoxide (DMSO)(Sigma-Aldrich) and added to the quintuplicate wells for 24 h. OD450 nm was detected using a microplate reader (M1000 PRO, TECAN). These experiments were performed in quintuplicate and repeated twice.

### Quantitative real-time PCR (qPCR)

RNA Trizol reagent (9109, Takara, Dalian, China) was used to extract total RNAs. Reversed-transcription was performed as described before [53].

### Immunohistochemistry (IHC)

The study was carried out in line with International Ethical Guidelines for Biomedical Research Involving Human Subjects (CIOMS). The tissue microarrays were purchased from Superbiotek (Shanghai, China), involving 15 normal cervical epithelial, 51 cervical intraepithelial neoplasia (CIN) and 95 CC, which were performed for immunohistochemistry (IHC) and IHC score analysis. The protocol was performed as previously described [53]. PRKDC (1:100; ab32566; Abcam),

IPO4 (1:100; ab181037; Abcam) were detected using the corresponding primary antibodies. Images of all the sections were taken using a fluorescence microscope (Carl Zeiss, Oberkochen, Germany). The total score was calculated based on the intensity of the cytoplasmic staining (0 = no staining, 1 = weak staining, 2 = moderate staining, and 3 = strong staining) plus the proportion of stained tumor cells (0 = 0%, 1 = 1–10%, 2 = 11–50%, 3 = 51–80%, and 4 = 81–100%), as blindly evaluated by two pathologists, independently. Tumors with scores ≥5 were classified into the high expression group, whereas ≤ 5 into the low expression group, which was applied before [52].

### Cell apoptosis assay

A density of 1 × 10^6^ target cells was plated into six-well plates and treated with CDDP (3 μg/ml), NU7026 (2 μM) or 0.1% DMSO as a control for 24 h. They were then labeled with annexin-V-fluorescein isothiocyanate and propidium iodide (BD Biosciences, Franklin Lakes, NJ, USA) as previously described [54].

### Immunofluorescence (IF)

CC cells were seeded in 8-well chambers (Ibidi, Germany) at a density of 3000 /well. And CC cells were treated with 6 μg/ml CDDP for 1 h for DNA double-strand break staining (γH2AX fluorescence). Target cells were then fixed with 4% polyformaldehyde for 30 min, permeabilized with 0.1% TritonX-100 for 10 min and blocked with 10% BSA for 1 h at room temperature. Blocked cells were incubated overnight with primary antibodies against CEBPD (1:25; sc365546; Santa Cruz), IPO4 (1:50; ab181037; Abcam), γH2AX (1:50; ab2839; Abcam) at 4 °C and then labeled with Alexa Fluor-594-conjugated secondary antibody (1:200) for 1 h at room temperature. While the nuclei were stained for 2 min with DAPI (Sigma, USA). Confocal microscopy (LSM 510, META Laser scanning microscope, Zeiss) was used to acquire images. γH2AX fluorescence was quantitated using ImageJ (NIH, Bethesda, MD).

### Terminal deoxynucleotidyl transferase (TdT) dUTP nick-end labeling (TUNEL) assay

A TUNEL kit (Roche, Basel, Switzerland) was used to quantify the proportion of apoptotic cells in tissue sections from the xenograft tumors. We performed this assay following the protocols as the previous study [54].

### Chromatin immunoprecipitation (CHIP) assay

ChIP assays were done as previously reported [55]. Antibodies against CEBPD (1:25; sc365546; Santa Cruz) and PRKDC (1:1000; ab32566; Abcam) were used for IP. The primers were listed in Supplementary Table 2.

### Luciferase reporter assay

Luciferase activity assays were carried out as described previously with some modifications, luciferase plasmids containing wild-type and 3 mutant PRKDC promoters were constructed in the pGL3B vectors with or without CDDP (1 μg/ml for 24 h). PRKDC promoters with plasmid CEBPD overexpressed SiHa cells were co-transfected with pGL3B. Luciferase activity was measured with a Dual-Glo luciferase assay kit (E290, E294, E2980, Promega, Madison, WI, USA). The sequences of primers the mutant constructs were listed in Supplementary Table 3.

### Co-immunoprecipitation (Co-IP) assay

Protein A/G beads were added to the cell lysate with three washes, then beads-lysate complexes were mixed with anti-CEBPD (10 μg; sc-365546; Santa Cruz) or anti-IPO4 antibodies (10 μg; ab181037; Abcam) and rotated overnight at 4 °C with IgG (Abcam, ab172730) as a negative control. Immunoprecipitates were then collected by centrifugation at 3000 × *g* for further western blotting.

### Western blotting and protein extracts

Western blot was performed as previously described [53]. Antibodies used were rabbit-anti-PRKDC (1:1000; ab32566; Abcam), anti-IPO4(1:1000; ab181037; Abcam), anti-Tubulin (1:3000; ab0049; Abways), anti-laminB (1:5000; ab0054; Abways), mouse anti-CEBPD (1:500; sc365546; Santa Cruz). Notably, PRKDC (450KD) protein was used 6% precast gels with some modifications. Boiling cell lysates were resolved on 6 % precast gels at 150 v for 40 min, then were transferred to a PVDF membrane (Millipore Sigma) using eBlot ® L1 wet transfer system (GenScript, Piscataway, NJ) for 15 min. While others (30–130KD) were used 10% precast gels at 150 v for 40 min and transferred with eBlot ® L1 wet transfer system for 11 min. The Thermo Scientific NE-PER Nuclear and Cytoplasmic Extraction Kit were used to extract the separate cytoplasm and nuclear. Cytoplasmic and nuclear fractionation was done according to the methods described [56]. Quantitative analysis of protein concentration was calculated using ImageJ (National Institutes of Health).

### Mouse xenograft model

Animal experiments were approved by the Institutional Animal Care and Use Committee of East China Normal University [57]. A total of 1 × 10^7^ SiHa cells stably expressing shNC, shPRKDC-1, shCEBPD-1, shIPO4-1 (suspended in 0.1 ml PBS) were respectively subcutaneously injected into 5-week-old nude female mice as described above. Once mice born visible tumors (100 mm^3^), mice were randomly assigned into separate groups (*n* = 5 per group), respectively: (1) shNC+saline groups, (2) shNC+CDDP groups, (3) shPRKDC+saline groups, (4) shPRKDC+CDDP groups, (5) shCEBPD+saline groups, (6) shCEBPD+CDDP groups, (7) NU7026 groups, (8) NU7026+CDDP groups according to the treatment of saline, CDDP or NU7026. CDDP was administered at a concentration of 5 mg/kg by intraperitoneal injection every 3 days, whereas NU7026 was 25 mg/kg every 3 days. All the control groups were taken the same since all the mouse xenograft experiments were done in the same time. Tumor volumes were measured every week up to 6 weeks. Tumor volumes were calculated using the formula: volume = (length × width^2^) × 0.5. All mice were killed at day 42, and the xenografts were stripped out and weighed for further analysis.

### Statistical analyses

Data are depicted as mean ± standard deviation (SD) or standard error of mean (SEM). Statistical analyses and graphical images were done using SPSS 22.0 (Chicago, USA) and GraphPad Prism 7.0 for Windows (San Diego, USA). Comparisons between groups were performed by student’s *t* test or one-way ANOVA test. A two-tailed *p* < 0.05 was considered statistically significant.

## Supplementary information

Supplementary Material

Supplementary Fig. 1

Supplementary Fig. 2

Supplementary Fig. 3

Supplementary Fig. 4

Supplementary Fig. 5

Supplementary Fig. 6

Supplementary Fig. 7
