# Supplementary material for: Inhibiting Importin 4-mediated nuclear import of CEBPD enhances chemosensitivity by repression of PRKDC-driven DNA damage repair in cervical cancer
Source: Oncogene. 2020 Jul 13;39(34):5633–48. doi: 10.1038/s41388-020-1384-3 (PMC7441007; doi:10.1038/s41388-020-1384-3)
Supplement: Supplementary file 1 — Supplementary Material [file 41388_2020_1384_MOESM1_ESM.docx]

**hSupplementary Figure 1.** ***PRKDC* is upregulated in CC and implicated in tumorigenesis.**

Analyses of mRNA expression *of PRKDC* in normal cervical tissues and CC in the published datasets (**A**) GSE7410 (**B**) GSE9750 (**C**) GSE7803. (**D**) GSE6791. (**E****)** Comparison of mRNA expression of *PRKDC* among T1，T2，T3，T4 in CC in TCGA datasets. (**F)** Comparison mRNA expression of *PRKDC* among Stage I, II, III, IV in CC in TCGA datasets. (Student's *t*-test; one-way ANOVA test). * *p* < 0.05, ** *p* < 0.01, and ****p* < 0.001.

**Supplementary Figure 2.** **Knockdown of PRKDC** **confers CDDP sensitivity in C4I cell *in vivo*.**

**(A)** *PRKDC* expression was examined by qPCR in different CC cell lines (C33A, HeLa, Ms751, CaSki, C4I, SiHa).

**(B)** PRKDC level was examined by Western blotting in different CC cell lines.

**(C)** Cell viability assay showing the sensitivity to CDDP in C4I /shPRKDC-1 and control shRNA. Refer to Fig.1I.

**(D and E)** Apoptosis assay staining the effect of 3 μg/ml CDDP and serum-free medium for 24 hours on the percentages of apoptotic C4I cells.

(**F and G**) Immunofluorescence showed the DNA damage induced by the 6μg/ml CDDP for 1 hour in C4I cell. Scale bar indicated 50 μm. γH2AX was shown by green fluorescence, and the [cell nuclei](https://www.sciencedirect.com/topics/medicine-and-dentistry/cell-nucleus) were stained with [DAPI](https://www.sciencedirect.com/topics/medicine-and-dentistry/dapi) (blue fluorescence). The percentage of cells with predominantly nuclear γH2AX was analyzed. *n* = 3 randomly chosen fields. Values are mean ± SD. (Student’s *t*-test). * *p* < 0.05, ** *p* < 0.01 and *** *p* < 0.001.

**Supplementary Figure 3. *In vivo* tumor pictures.**

A total of 1 × 10^7^ SiHa cells stably expressing shNC, shPRKDC-1, shCEBPD-1, shIPO4-1 (suspended in 0.1 ml PBS) were respectively subcutaneously injected into 5-week-old nude female mice. Once mice born visible tumors (100 mm^3^), mice were randomly assigned into separate groups (n = 5 per group) according to the treatment of saline, CDDP or NU7026. Control groups (saline and CDDP) were taken the same, since all the *in vivo* tests were performed at the same time.

**Supplementary Figure 4.** **CEBPD attenuates CDDP-sensitivity in C4I cell.**

**(A**) Apoptosis analysis was calculated in C4I/shCEBPD-1 and control shRNA cells. Refer to Fig.1H.

**(B**) Cell viability was analyzed in C4I/shCEBPD-1 and control shRNA cells. Refer to Fig.1I**.**

**(C**) γH2AX assay and quantitative analysis were performed in C4I/shCEBPD-1 and control shRNA cells. Refer to Fig.1H. Values are mean ± SD. (student's *t*-test). * *p* < 0.05, ** *p* < 0.01 and *** *p* < 0.001.

**Supplementary Fig. 5.** **IPO4 colocalizes with CEBPD.**

Colocalization of IPO4 and CEBPD demonstrated by IF, confocal microscopy using appropriate filters. Merged images of CEBPD (green), DAPI (blue) and IPO4 (red) are shown.

**Supplementary Figure 6. IPO4-knockdown improves CDDP-sensitivity in C4I cell.**

**(A**) Apoptosis analysis was performed in C4I/shIPO4-1 and control shRNA cells. Refer to Fig.1H.

**(B**) Cell viability was analyzed in C4I/shIPO4-1 and control shRNA cells. Refer to Fig.1I.

**(C**) Quantitative γH2AX assay was calculated in C4I/shIPO4-1 and control shRNA cells. Refer to Fig.1J.Values are mean ± SD. (Student's *t*-test). ***p* < 0.01 and *** *p* < 0.001.

**Supplementary Figure 7.** ***IPO4* is upregulated in CC and implicated in tumorigenesis.**

**(A)** mRNA of *IPO4* was compared with normal tissue and CC tissue in GSE9750. (Student's *t*-test).

**(B and C)** IPO4 expression was compared based on T1, T2, T3, T4 or Stage I, II, III, IV from TCGA datasets. one-way ANOVA test. * *p* < 0.05 and ** *p* < 0.01.

| **Supplementary Table 1.** The correlation between karyopherins and PRKDC before and after chemotherapy | | | | |
| --- | --- | --- | --- | --- |
| Correlation with PRKDC | | | | |
| Karyopherins | | Prior to chemotherapy | Post chemotherapy | |
| KNPA1 | 0.254 | | 0.518** |  |
| KNPA4 | | 0.199 | 0.525** | |
| IPO4 | | 0.211 | 0.464** | |
| XPO6 | | 0.236 | 0.456** | |
| TNPO3 | | 0.080 | 0.429** | |
| KNPA3 | | 0.252 | 0.399* | |
| XPO2 | | 0.604** | 0.888** | |
| XPO7 | | 0.722** | 0.809** | |
| XPOT | | 0.563** | 0.679** | |
| IPO11 | | 0.511** | 0.664** | |
| IPO5 | | 0.232 | 0.271 | |
| IPO7 | | 0.429** | 0.402* | |
| IPO8 | | 0.461** | 0.307 | |
| IPO9 | | -0.044 | 0.108 | |
| IPO13 | | -0.066 | -0.001 | |
| KPNA2 | | 0.601** | 0.578** | |
| KPNA5 | | 0.526** | 0.423** | |
| KPNA6 | | 0.762** | 0.580** | |
| KPNB1 | | 0.828** | 0.722** | |
| TNPO1 | | 0.357* | 0.059 | |
| TNPO2 | | 0.08 | 0.191 | |
| XPO1 | | 0.640** | 0.633** | |
| XPO5 | | 0.542** | 0.439** | |

*P<0.005，**P<0.01，***P<0.001

**Supplementary Table 2.** Primers used in the ChIP-PCR for indicated genes

|  | Forward Primer Sequence (5’-3’) | Reverse Primer Sequence (5’-3’) |  |
| --- | --- | --- | --- |
| 1 | AGCCGCAGCAGGGAGCAACGCAC | CACCCAAACTACCTCCGCAGGTC |  |
|  |  |  |  |
|  |  |  |  |
| 2 | CCCGGAGGCGGAGCCACGTACGA | CTCCCGGCTTCCCTGCCCTGCTC |  |
|  |  |  |  |
|  |  |  |  |
| 3 | GAGCCACCGCACCGTGCCACATA | TTTAGCAATGCCTGGGTGGGAAC |  |
|  |  |  |  |
|  |  |  |  |
| **Supplementary Table 3.** The sequences of primers the mutant constructs（Highlight areas are the mutation sequences）  **Sequence of mutated PRKDC promoter region**  Mutant 1  CGGATCAGTTGATGACCGGCCAGGGCAGCACCGCAGCGGTCCGCAGCGGA | | | |
| CAAGGTCTCCTGCAGCCGCAGCAGGGAGCAACGCACACCGGCTCCGGAG | | | |
| CCCGCCATGCCGCCGAGTCCCGCTCCCGCGCGTGCGCCCGCTCGGCCCGG | | | |
| ACCCGGAAATGCCCCTACGCGCGGAGGCGGGGCTGCGGGGCGCGGCGGC | | | |
| AGGAACTTTCCCGGGGACCCCTGCGGGAAGGGGATTACAGTAAGCGCGCC | | | |
| TCTTTGGCCCGAATCAACATGGAAACCTAAGGAAAAACGTCTGACCTGCG | | | |
| GAGGTAGTTTGGGTGGCTACTTGGTGTTGGACTTGGCTAAATAAATACCAG | | | |
| AGGCAGATAAAACAAGCGAGGGGCCTAGGTCAAGAGTTCCAAGTTTGTTC | | | |
| CTCGATAAAAAACGACAAACGCGGTCGGGGGCGGTGATGCCCGCCTGCAG | | | |
| TCCCAACACTTTA | | |  |

Mutant **2**

| AAGTGAGAAATGCATCTGTAATGTCCTTATTAACCAAAAACGCCTAAGGGA | |
| --- | --- |
| TAAAGAAGCACACGCTCCCTAGCCAACGCTAGAGGAACACACCCCGAGAG | |
| CTGGTGCTGTAGGACCCAGTTTTCCCGCGAAAACGCTGCCGCGCAGGGGG | |
| TCAGACCATCTGGACCAAGGGGGGCCGAGCGAGGCCTACTTCTGGTTTAC | |
| GCACGGGCGCTGAAAGAAGCGGCACTGTCCCCCCCTGCCGATGCGCAGTG | |
| GCGCCTCCCGGAGGCGGAGCCACGTACGAGCGCCGCTGTGATTGGTGAGG | |
| CCCCGCCAGGGGCGGAGACGACCTTGCCGCCGGCGGGAACTCTGGGTCTC | |
| GCGGTTTGGGAGCGCTACTCGCCAGGTGGACTCGGAGTCCGCGAGCGTCG | |
| TCGGCAAGCGGCCGCCTTTCCACGGTAAGGATTAGCCGGCGGGGAGGGCG | |
| TGGCGCGGAGCCGACGGGAACGTCCGCGCTGCGGAGCAGGGCAGGGAAG | |
| CCGGGAGGCGGGCCCGGCCCGAGCTTGTCCTTGTCGCGCAGGTACTCCGA | |
| GCACTATGTCGTCCCCGGCGTCGACCCCGAGCCGCCGCGGCAGCCGGCGT | |
| GGAAGGGCCACCCCCGCCCAGACGCGTGAGTCCCCCGAGCCGGGCCCACT | |
| ACAGCCCCCGGCGCCGCCCCGTCTGCCCTCTCGCCGCAGCTGGCAGCGCT | |
| GGGTGGGTGCGCGGGACCCGGGCGCTCAGCCTCGGGCTGGGCGCTGCCGC | |
| TTGGTGCGCACAGACACCCACAGCAGGCTGTGGCCTGGGTGCTGCTTAATT | |
| CGATTGCCATTTGCCTCTGTTTGGTTTGGTTCAGTGGTGAGTCATAATGCCC | |
| CAAGGA |  |

Mutant 3

| GCTCGCCACCATGCCCAGTTAATTTTTGTATTTTTAGTAGAGACAAGGTTTC |
| --- |
| ACCATACTGGTCAGGTTGGTCTCCATCTCCTGACCTCAGGTGATCCAGCTG |
| CCACAGCCTCCCAAAGTGCTGGGATTACAGGCATGAGCCACCGCACCGTG |
| CCACATATAGCATCTAGTTTAAACTGTTTAAGTTATTCCTACACCATATTCAC |
| TACTAGACCCTCCGGAAGAAAATCTCACCTTTTAATAGGGCACAACATGGT |
| TTACTGCATACTCGTGTTTTAAAGAAGGAATACAGGAGATCCACATAATTAA |
| GTATTTGAATATTTAAAATGCTAAAAAGAGGCACTTTTACTACAAAGGGGA |
| AAAAGTTCCCACCCAGGCATTGCTAAAGAGTTAAAAGAACATGACTTCAAT |
| TACATTTCTTCAATTTTTCTCAAAGTAGGAACGGCAAATAGAGGGGTTTGG |
| AAAAGGTACTGGTGGGATTAGGCGAGATTCAGGTGAAAGAAAA |
