# Supplementary figures and images for: Inhibiting Importin 4-mediated nuclear import of CEBPD enhances chemosensitivity by repression of PRKDC-driven DNA damage repair in cervical cancer
Source: Oncogene. 2020 Jul 13;39(34):5633–48. doi: 10.1038/s41388-020-1384-3 (PMC7441007; doi:10.1038/s41388-020-1384-3)

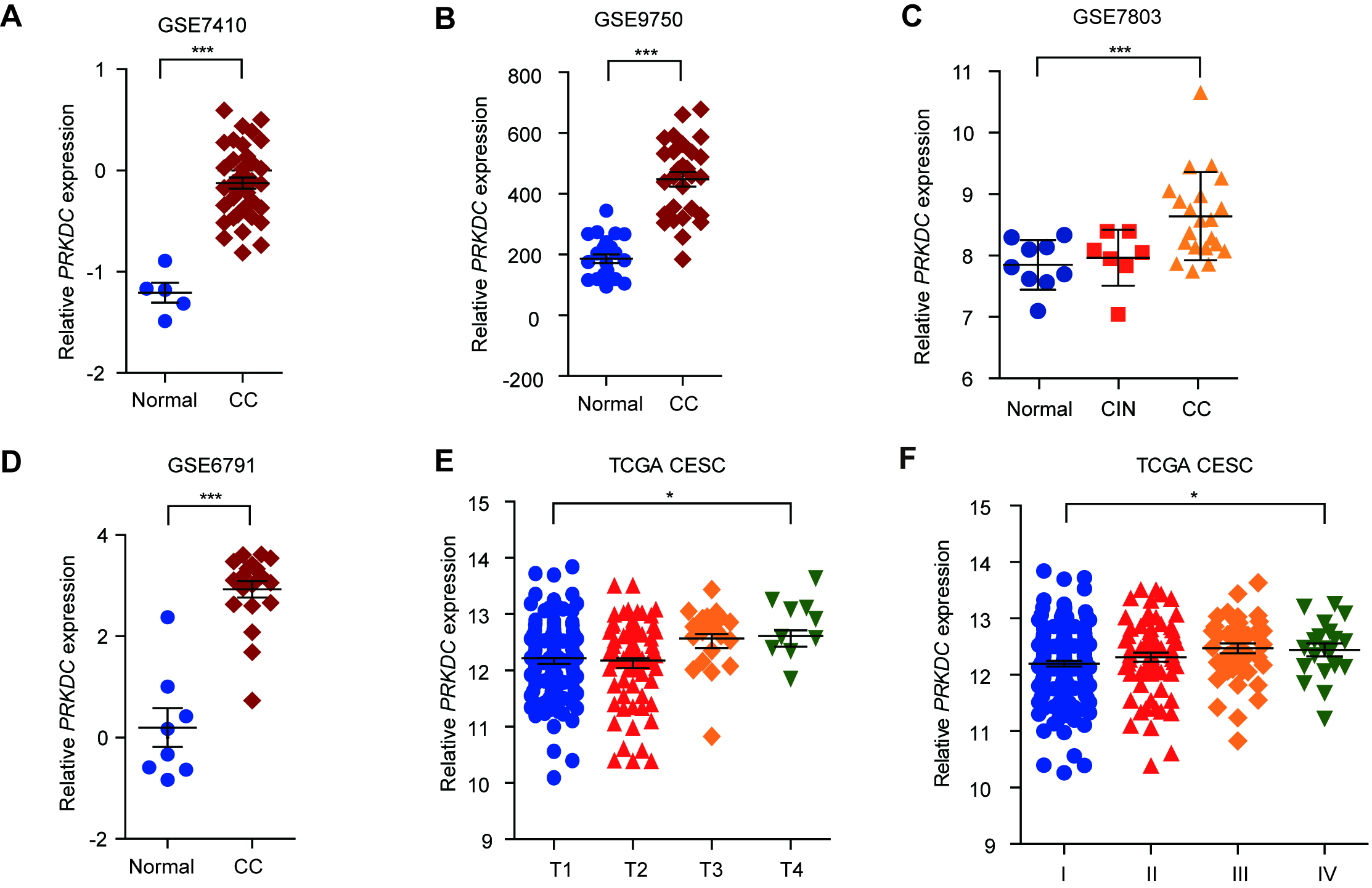

Supplement: Supplementary file 2 — Supplementary Fig. 1 [file 41388_2020_1384_MOESM2_ESM.tif]

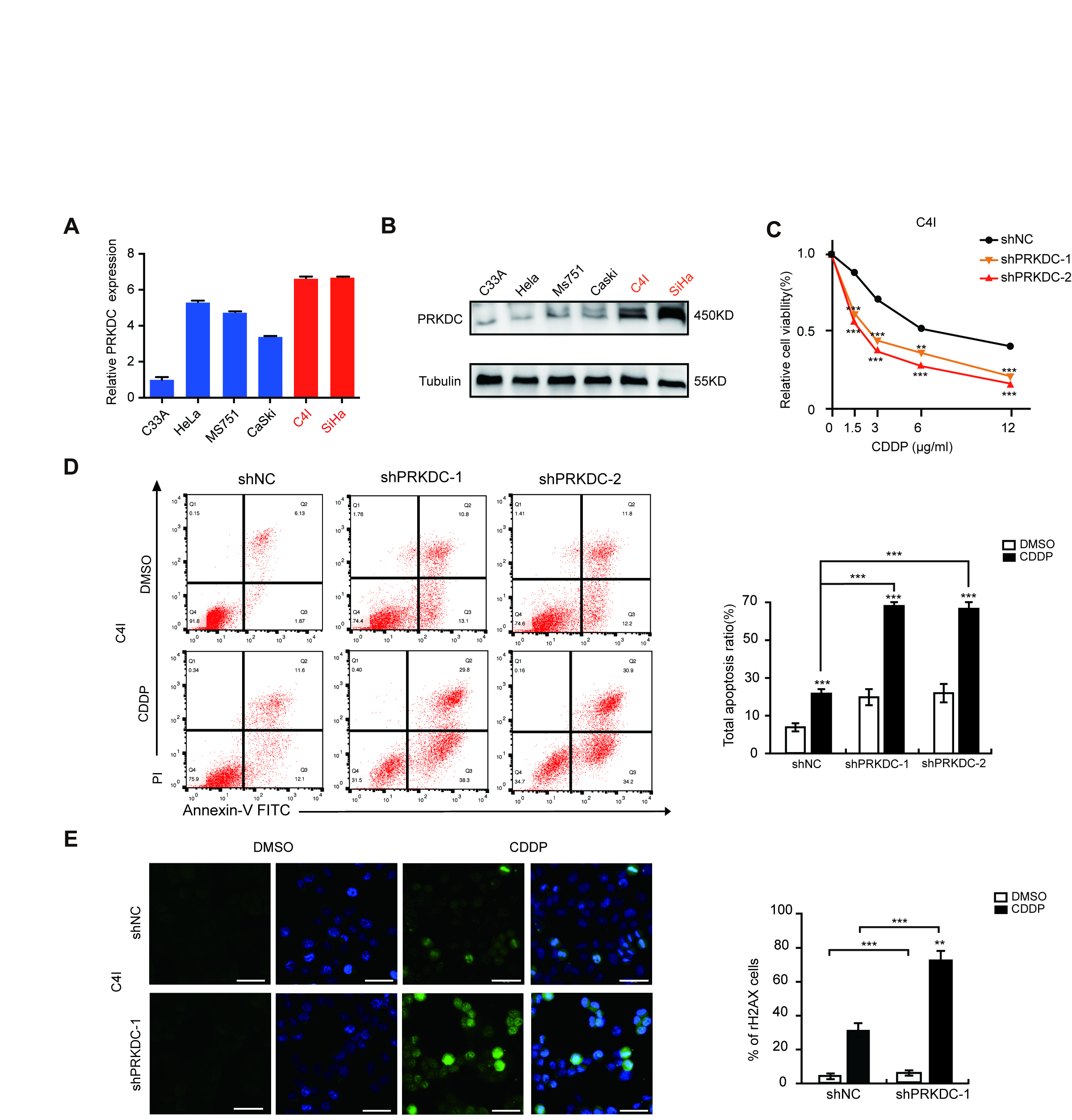

Supplement: Supplementary file 3 — Supplementary Fig. 2 [file 41388_2020_1384_MOESM3_ESM.tif]

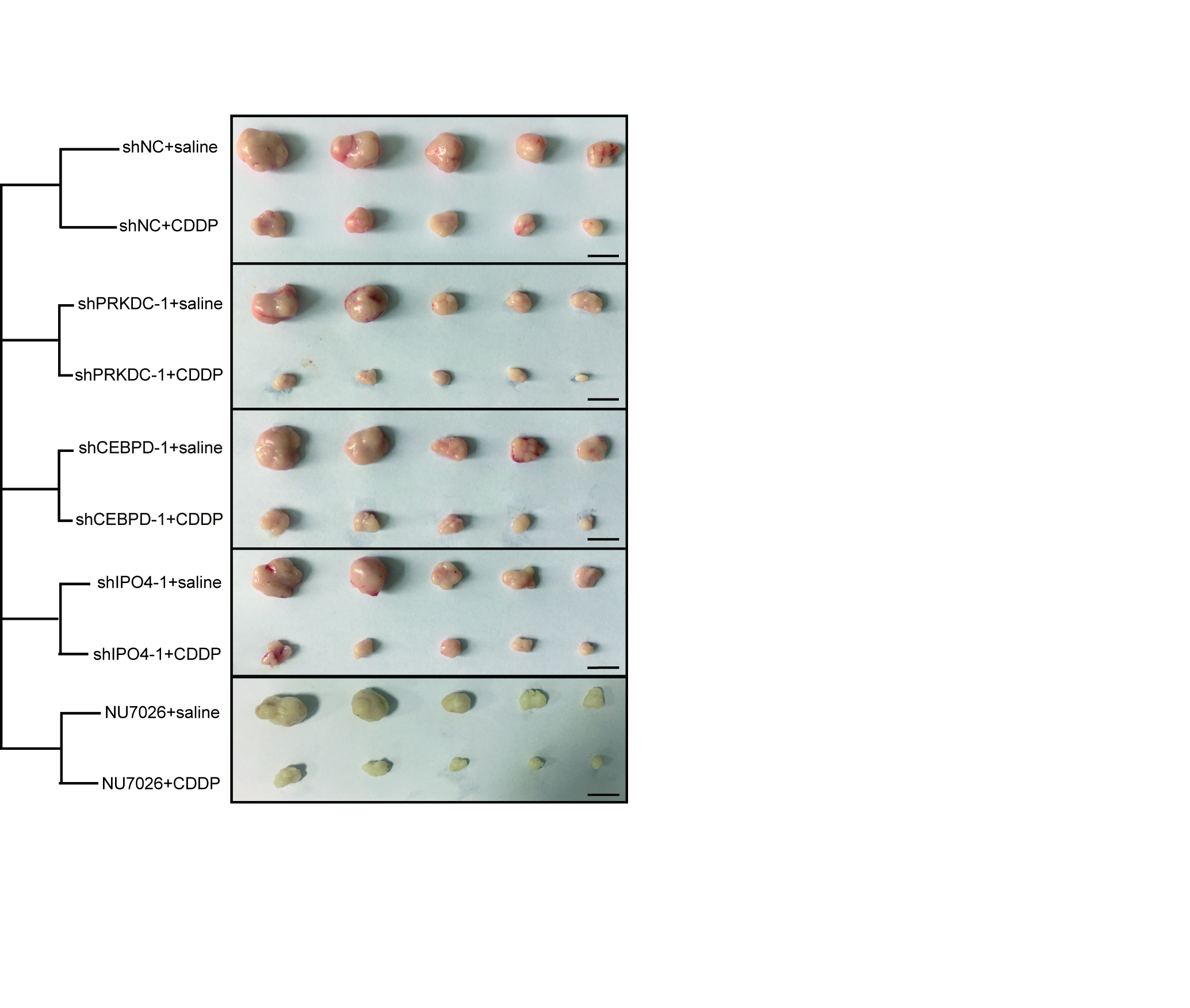

Supplement: Supplementary file 4 — Supplementary Fig. 3 [file 41388_2020_1384_MOESM4_ESM.tif]

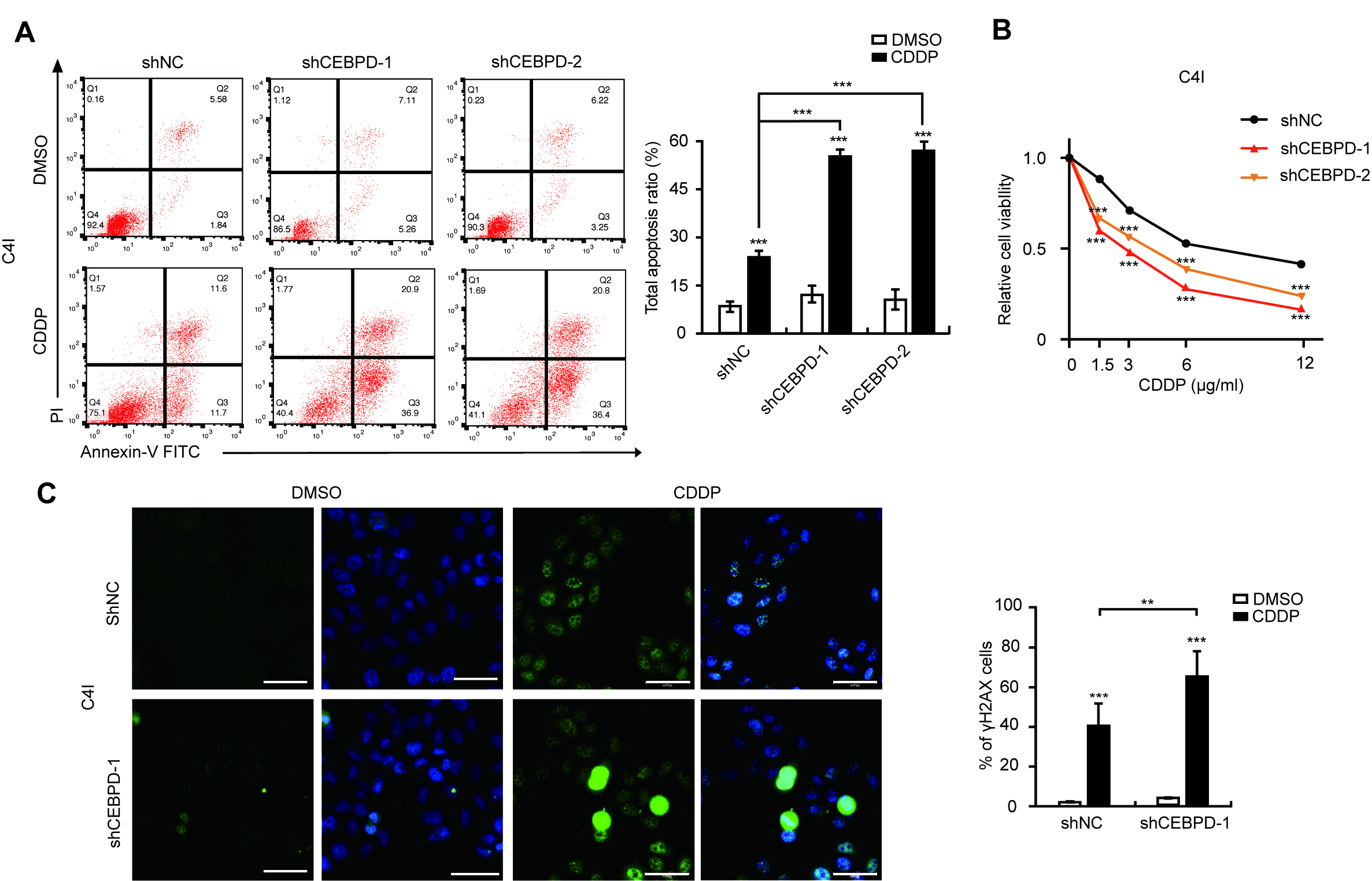

Supplement: Supplementary file 5 — Supplementary Fig. 4 [file 41388_2020_1384_MOESM5_ESM.tif]

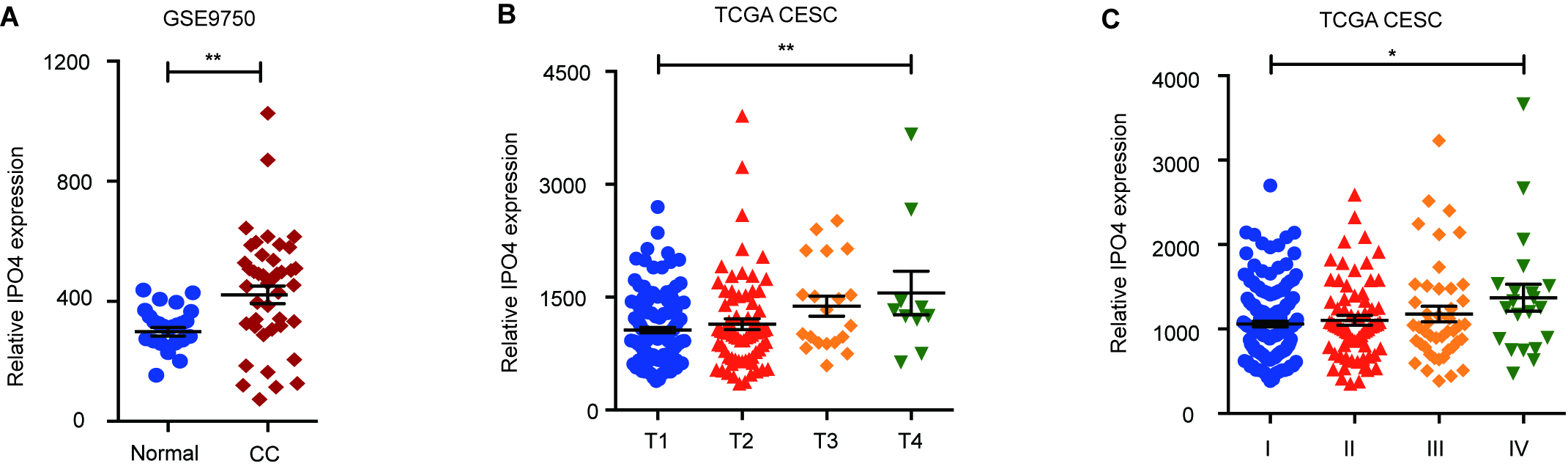

Supplement: Supplementary file 6 — Supplementary Fig. 5 [file 41388_2020_1384_MOESM6_ESM.tif]

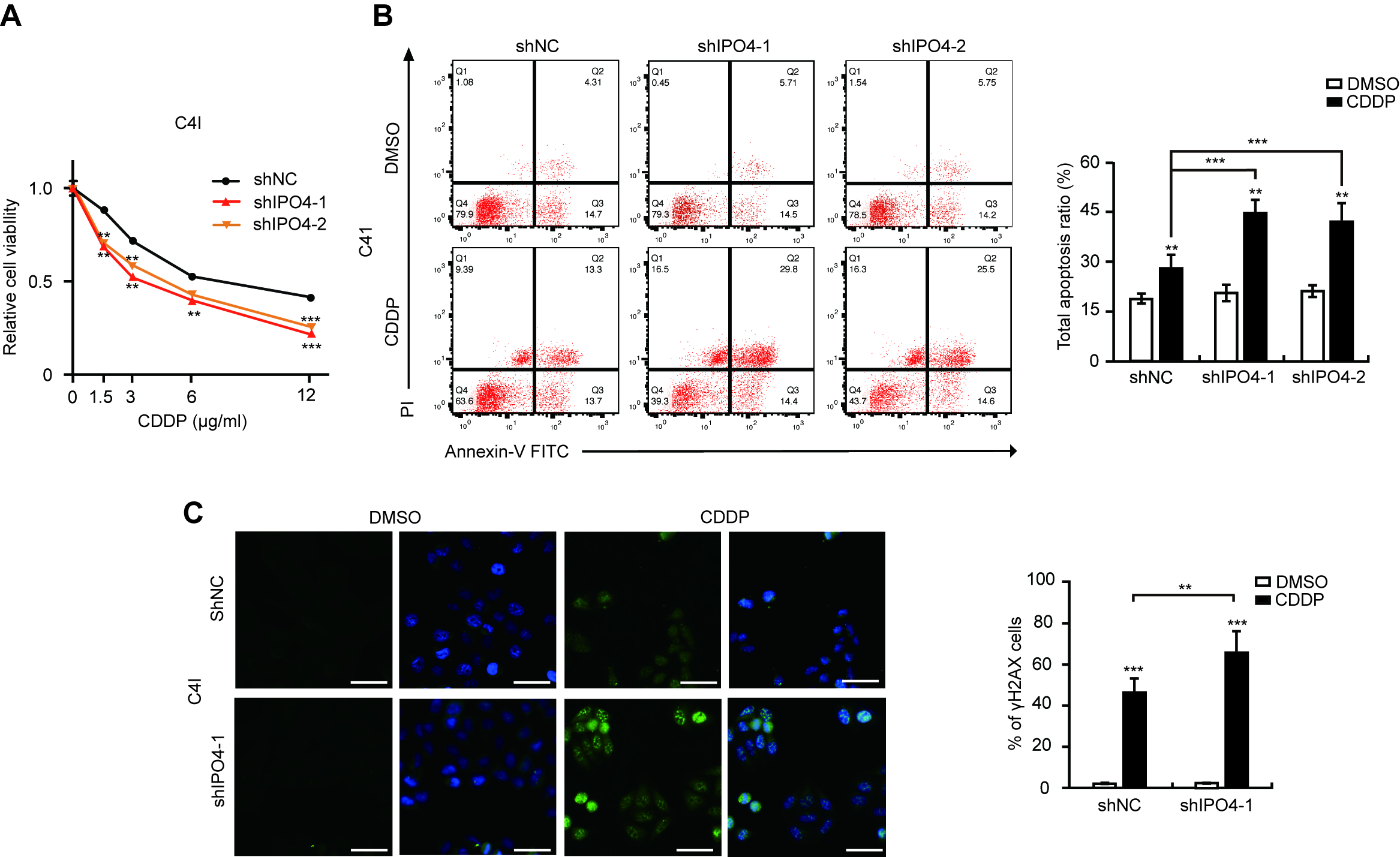

Supplement: Supplementary file 7 — Supplementary Fig. 6 [file 41388_2020_1384_MOESM7_ESM.tif]

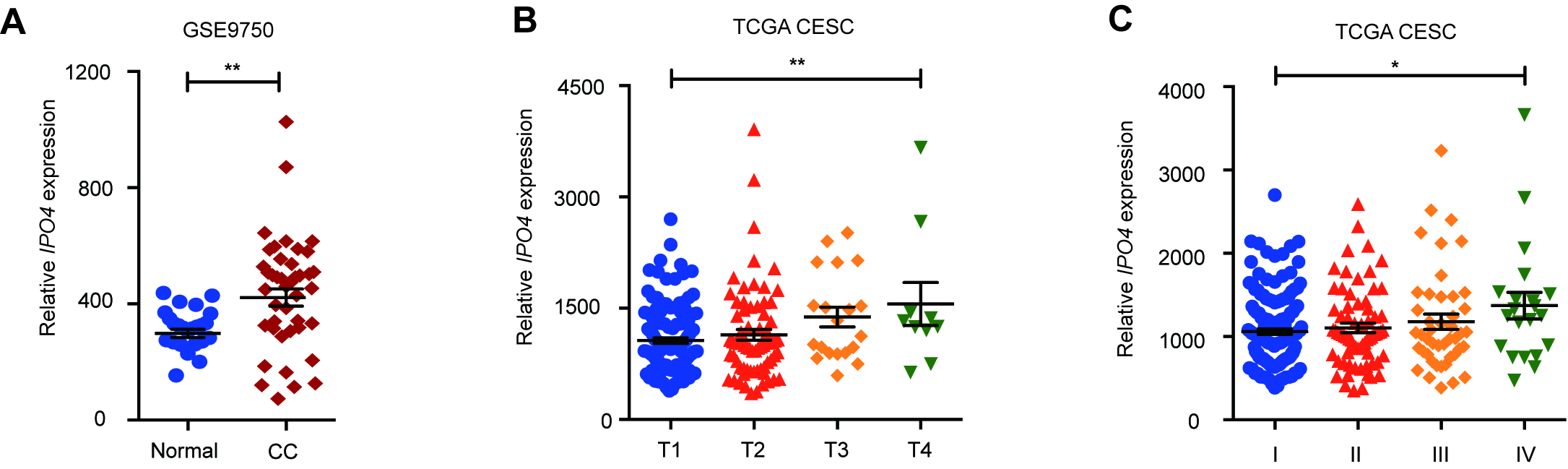

Supplement: Supplementary file 8 — Supplementary Fig. 7 [file 41388_2020_1384_MOESM8_ESM.tif]
